# The Impact of Mental Health Comorbidities on Unplanned Admissions for Physical Conditions: A Retrospective Observational Analysis

**DOI:** 10.3390/healthcare13070827

**Published:** 2025-04-04

**Authors:** Fabrizio Cedrone, Omar Enzo Santangelo, Vittorio Di Michele, Alessandro Catalini, Flavia Pennisi, Lorenzo Stacchini, Marco Fonzo, Vincenzo Montagna, Vincenza Gianfredi, Giuseppe Di Martino

**Affiliations:** 1 Hospital Management, Local Health Autority of Pescara, 65100 Pescara, Italy; fabrizio.cedrone@asl.pe.it; 2 Regional Health Care and Social Agency of Lodi, ASST Lodi, 26900 Lodi, Italy; omarenzosantangelo@hotmail.it; 3 Department of Mental Health, Local Health Autority of Pescara, 65100 Pescara, Italy; vittorio.dimichele@asl.pe.it; 4 Food Safety and Nutrition Unit, Macerata Local Health Authority, Via Annibali 31, 62100 Macerata, Italy; alecata@icloud.com; 5 Ph.D. National Programme in One Health Approaches to Infectious Diseases and Life Science Research, Department of Public Health, Experimental and Forensic Medicine, University of Pavia, 27100 Pavia, Italy; pennisi.flavia@hsr.it; 6 School of Medicine, Università Vita-Salute San Raffaele, 20132 Milano, Italy; 7 Department of Health Science, University of Florence, 50134 Florence, Italy; lorenzo.stacchini@uslnordovest.toscana.it; 8 Department of Community Healthcare Network, Azienda USL Toscana Nord Ovest, 56121 Pisa, Italy; 9 Hygiene and Public Health Unit, Department of Cardiac-Thoracic-Vascular Sciences and Public Health, University of Padova, 35131 Padova, Italy; marco.fonzo@unipd.it; 10 Hospital Management, Local Health Autority of Teramo, 64100 Teramo, Italy; vincenzo.montagna@aslteramo.it; 11 Department of Biomedical Sciences for Health, University of Milano, Via Pascal, 36, 20133 Milano, Italy; vincenza.gianfredi@unimi.it; 12 Department of Medicine and Ageing Sciences, “G. d’Annunzio” University of Chieti-Pescara, 66100 Chieti, Italy; 13 Unit of Epidemiology and Health Statistics, Local Health Autority of Pescara, 65100 Pescara, Italy

**Keywords:** unplanned hospital admissions, mental health comorbidities, deprivation index, hospital discharge records, public health, healthcare inequalities, socio-economic disparities, chronic diseases, retrospective study

## Abstract

**Background**: This study aimed to evaluate the impact of mental health comorbidities on unplanned hospital admissions (UHAs) in the Province of Pescara, Southern Italy, during 2015–2022. Mental health comorbidities are underreported in administrative data, yet their association with UHAs has significant public health implications. **Methods**: A retrospective observational design was used to analyze 59,374 hospital admissions extracted from hospital discharge records (HDRs). Admissions of patients under 18 years of age, deliveries, day admissions, and readmissions were excluded. Socio-economic deprivation was assessed using a standardized deprivation index. Multivariate logistic regression analyzed the association between UHAs and mental health comorbidities, adjusting for socio-demographic and clinical factors. **Results**: Of the 59,374 admissions, 43,293 (72.9%) were unplanned. Mental health comorbidities had a low prevalence (1552 cases, 2.6%) but were significantly more common in UHAs (3.4%) compared to planned admissions (0.4%, *p* < 0.001). UHAs were also associated with the female gender (OR = 1.10; 95% CI: 1.06–1.14), younger age categories, living in less deprived areas, two or more physical comorbidities (OR = 1.66; 95% CI: 1.56–1.75), and mental health comorbidities (aOR = 9.85; 95% CI: 7.74–12.55, *p* < 0.001). **Conclusions**: Mental health comorbidities significantly increase the risk of UHAs independent of socio-economic deprivation or physical comorbidities. These findings underscore the need for enhanced mental health management to reduce UHAs, improve patient outcomes, and address healthcare inequities.

## 1. Introduction

The rising prevalence of chronic conditions is one of the most relevant public health issues needing a global effort [1]. It is estimated that most people aged over 65 years live with multimorbidity (defined as the presence of more than one chronic condition), and the mean number of comorbidities increases with age [2,3]. Nevertheless, multimorbidity is not limited to older patients; in recent years some studies revealed that this phenomenon also affects adults and young adults [4]. Specifically, the management of physical illness in patients with comorbid mental diseases is a significant public health issue, particularly in high-income countries [5]. Nearly half of people with any mental disorder have comorbid medical problems, while a further 35% have undiagnosed medical conditions [6].

It is well known that patients with mental illness report a shorter life expectancy compared to patients without mental issues. Considering that this gap is mainly attributable to physical illnesses [7], mental illness can be seen as a risk factor for the development of chronic and lifestyle-related conditions [8,9]. Although depression and dementia are known risk factors for increased all-cause mortality [10], evidence of the association of other mental health conditions, such as anxiety and depression, with chronic physical conditions is still lacking. A possible explanation for this gap in knowledge could be the absence of many mental conditions in most of the available clinical prediction scores. For example, most used indices, such as Charlosn’s Comorbidity Index or Elixhauser Comorbidity Index, do not include several mental conditions in their computation.

The management of both mental and physical conditions is challenging. Patients with mental conditions are known to face challenges in accessing healthcare [8,9,11]. Furthermore, they seem less likely to be offered health-promotion interventions and less prone to seek medical attention. They also exhibit lower compliance with lifestyle interventions and in the consumption of prescribed medications [12]. The combination of these factors leads to poor disease management in the primary care setting that, in turn, frequently results in unplanned hospital admissions (UHAs). Access to healthcare can be categorized as either planned or unplanned. At the hospital level, planned access typically follows a clinical pathway and is initiated by a general practitioner or specialist for diagnostic or therapeutic interventions. In contrast, unplanned admissions are usually triggered by the sudden onset of an acute medical condition or the deterioration of a pre-existing disease. In such cases, patients access hospital care—often with or without a medical referral—through the emergency department. UHA may indicate shortcomings in the management of diseases within non-hospital settings. Therefore, UHA rates are important indirect indicators of primary healthcare quality. Reducing UHA is crucial for improving patient outcomes. In fact, recurrent hospital stays, particularly among patients with multimorbidities, increase the risk of hospital-acquired infections. Moreover, UHAs place a significant financial burden on healthcare systems. One study estimated that 32% of the total costs associated with index admissions can be attributed to UHAs [13].

Given their challenges in accessing healthcare, people with comorbid mental health conditions could represent a subgroup of patients on which public health intervention can focus to limit the occurrence of UHA. In order to do that, it is imperative to study the phenomenon and provide reliable estimates on the association between comorbid mental health conditions and UHA that can support programs.

In Italy, only a few studies investigated the impact of comorbid mental health conditions on UHA for physical conditions. For this reason, the aim of this study was to evaluate the impact of mental health conditions on UHA in the Province of Italian Southern Region, by evaluating the hospital discharge record from 2015 to 2022.

## 2. Materials and Methods

This was a retrospective observational study performed in the Local Health Authority (LHA) of Pescara, a Province of the Abruzzo region with a population of approximately 320,000 inhabitants in the period 2015–2022. The LHA has three hospitals: a tertiary referral hospital (hub) and two secondary hospitals (spokes). Data were collected from the LHA registry of hospital discharge records (HDRs). The HDRs include a large variety of data regarding patients’ demographic characteristics and hospitalization such as gender, age, and other information such as admission and discharge date and the discharge type, which also includes death. The HDRs also include information about the diagnoses that led to hospitalization or that are concurrent including complications (a maximum of six diagnoses, one principal diagnosis, and up to five comorbidities) and a maximum of six procedures or interventions that the patient underwent during hospitalization. Diagnoses and procedures were coded according to the International Classification of Disease, 9th Revision, Clinical Modification (ICD-9-CM), the National Center for Health Statistics (NCHS), and the Centers for Medicare and Medicaid Services External, Atlanta, GA, USA.

Using an algorithm proposed by Quan et al. [14], comorbidities were coded according to the Elixauser Comorbidity Index. We constructed a variable for physical health using a simple unweighted count of physical health conditions (0, 1, and ≥2). With the 4 mental health comorbidities (depression, psychosis, alcohol abuse, and drug abuse) we constructed a binary variable (0, 1) indicating the presence or absence of one of the diagnoses. Only mental conditions included in the Elixauser Comorbidity Index were considered.

The deprivation index (DI) used in this study is the one proposed by Caranci et al. [15,16,17]. The DI was applied at the municipality level in order to combine it with the HDRs in which the municipality of residence is recorded. DI is calculated as the sum of standardized indicators of low education level, unemployment, rented housing, single parent family, and housing density, and represents a proxy of the socio-economic level of each municipality. The DI was divided into classes based on population quintiles, ranging from the most deprived (5th quintile) to the least deprived (1st quintile). The latter identifies the 20% of the population with the lowest index values. The index is widely used to facilitate the identification of policies and resources aimed at reducing socio-economic inequalities [18].

The dependent variable was constructed through a careful selection of HDRs. Psychiatric hospitalizations, day hospitalizations, and hospitalizations related to childbirth were eliminated. Only the hospitalizations of adult patients were taken into consideration and, given the long study period, only the first hospitalization was selected. The UHAs were selected through a specific field present in the HDRs.

The qualitative variables were summarized as frequency and percentage. We constructed a logistic regression model with hospital admission (planned or unplanned) as the outcome variable. The independent variables included sex, age in categories (18–44, 45–64, 65–79, and ≥80), DI in the five deprivation classes, the number of physical disabilities (0, 1, and ≥2), and the presence or absence of a mental health diagnosis as described above. We then calculated predicted probabilities for a UHA to hospital from these models.

## 3. Results

**Figure 1:**
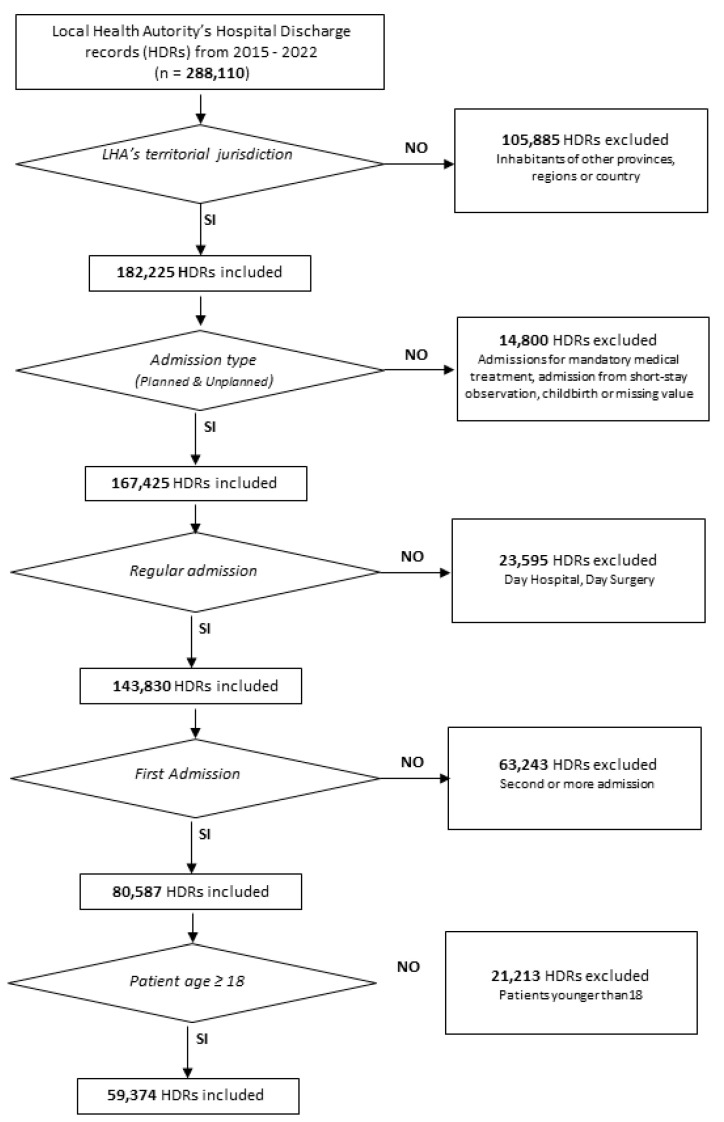
Flowchart of the hospital discharge record selection process.

In total, 288,110 hospital admissions were performed in the Province of Pescara during the years 2015–2022. Of these, 105,885 admissions were for patients residing in other Italian provinces and they were excluded from the analysis. Admissions of patients younger than 18 years, deliveries, day admission, and re-admissions were also excluded from the dataset. Finally, 59,374 admissions were included in the analysis, as reported in Figure 1. The admitted patients were mainly female (33,569, 56.5%), aged between 18 and 34 years (18,001, 30.3%), living in the most deprived area (34,489, 58.1%), and without physical comorbidities (35,357, 59.5%). Only 1552 patients (2.6%) reported a mental health condition.

The majority of the included hospitalizations were unplanned (43,293, 72.9%), and 1482 of them were referred to patients with a prevalent mental health comorbidity (3.4%). Among the planned hospitalizations, only 70 (0.4%) involved patients with mental health conditions. The characteristics of the included patients are reported in Table 1.

Logistic regression analysis identified several factors significantly associated with unplanned admissions. Female gender (OR: 1.10; 95%CI 1.06–1.14; *p* < 0.001), younger age groups (compared to those aged more than 80 years), living in less deprived areas, and reporting two or more comorbidities (OR: 1.66; 95%CI 1.56–1.75; *p* < 0.001) were all significant risk factors for unplanned admissions. In addition, reporting a mental health comorbidity was also strongly associated with unplanned admission (OR: 9.85; 95%CI 7.74–12.85; *p* < 0.001). The results of the logistic analysis are reported in Table 2.

**Figure 2:**
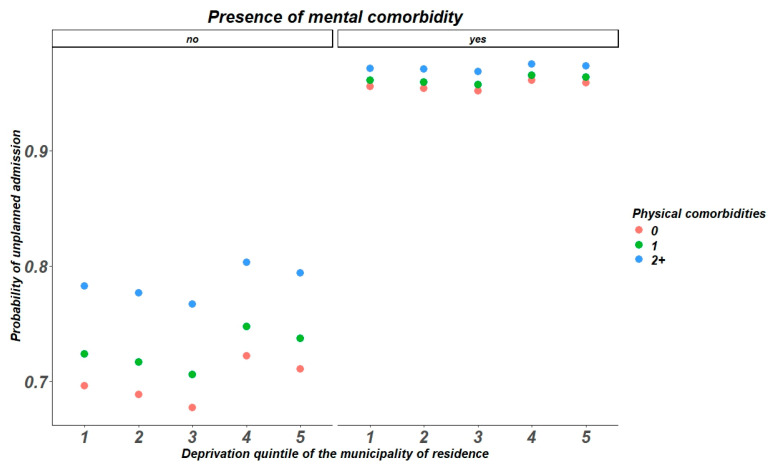
Probability of unplanned admissions accordingly to Deprivation Index, physical comorbidities and mental health conditions.

The probability of unplanned admissionis directly affected by the number of comorbidities and DI among patients without mental comorbidities. In the other hand, the presence of a mental condition enhance the probability of unplanned admissions, without differences across DI quintiles and number of comorbidities, as reported in Figure 2.

## 4. Discussion

The present study aimed to evaluate the impact of mental health comorbidities on unplanned hospital admissions in an entire Province of Southern Italy. During the years 2015–2022, a total of 59,374 eligible hospital admissions were recorded, of which 43,293 (72.9%) were unplanned. Mental comorbidities had a low prevalence among the considered admissions (1552 cases, 2.6%), but they were more frequent among patients with unplanned admission (1482 cases, 3.4%) compared to planned admissions (70 cases, 0.4%) (*p* < 0.001). Furthermore, socio-economic deprivation status was differently distributed by type of admission; in particular, patients in the most deprived areas were more frequently hospitalized (34,489, 58.1%), particularly for unplanned admissions (25,524, 59.0%) compared to planned admissions (8965, 55.7%). Multivariate analysis showed that mental comorbidities were significantly associated with unplanned admissions for any reason (aOR = 9.85, 95%CI 7.74–12.55, *p*-value < 0.001). Previous studies from other countries tried to evaluate similar associations. In particular, Geier et al. [19] evaluated the impact of psychiatric comorbidities on emergency general surgery patients. This study showed how psychiatric conditions were particularly prevalent among hospitalized patients. The greatest strength of that study was the direct assessment of mental health conditions via structured clinical diagnostic interviews, leading to a higher reported prevalence of mental disorders. Conversely, the present study evaluated the prevalence of mental comorbidities based on administrative data, where the diagnostic code of some comorbidities can be underreported. Similarly, a previous study by Pilowsky et al. [20] performed among ICU survivors found that pre-existing mental condition was a significant predictor of unplanned hospitalization (OR 1.57, 95% CI: 1.39–1.76). A similar prevalence in pre-existing mental comorbidities was reported by Okuma et al. (5% of patients admitted in a Japanese tertiary hospital from 2017 to 2020), confirming also the significant association between mental comorbidities and unplanned admissions (OR: 2.40, 95% CI 1.97–2.94; *p* < 0.001). The reasons for this significant association include the underestimation of the physical condition of patients with comorbid mental illness [21] and delays in diagnostic procedures or visits due to social or economic barriers [22]. This latter explanation is further supported by the significant association between unplanned admissions and the deprivation index. Margins analysis showed that the presence of a mental illness increased the odds of unplanned hospitalizations, independently of the number of comorbidities or deprivation index. These results emphasize the need to improve mental illness management, both to enhance psychiatric outcomes and to prevent the worsening of other physical comorbidities that lead to hospitalizations. A deep examination of primary care patients is needed to find undiagnosed patients with mild mental illness and to improve the management of psychiatric patients in the emergency department. Additionally, mental health comorbidities can increase the length of hospitalization, as demonstrated in the study by Carter et al. [23]. Among heart failure patients, those with mental comorbidities such as depression, bipolar disorders, or dementia experience an average increase of 3.3 days in hospitalization length compared to those without such conditions. Similar results have been reported by other authors such as Gentil et al. [24], and Hill et al. [25], who found that mental disorders not only increase the length of hospitalization but also raise the likelihood of readmission, particularly in the short and medium term. In addition, as demonstrated by Protty et al. [26], in acute coronary syndrome patients the mean length of hospitalization in patients without psychiatric comorbidities was 9.78 days, while it increased up to 14.72 days if the patient had received any psychiatric diagnosis, up to 20.87 days in case of dementia (20.87) and up to 13.41 days in case of mood disorders. Also, the disease outcomes were worse by 18% in those who had psychiatric comorbidities and the mortality rate in the latter was always higher by 26% compared to those who had no mental health problems [26].

As demonstrated in a recently published Canadian study [27], it is well known that patients with mental health problems frequently use emergency departments but their frequent use of the emergency room and adverse outcomes could derive from unmet needs and suboptimal care; therefore, as verified by Brown et al. [28], it would be appropriate to take charge of the psychiatric patient by carrying out mental health visits after a hospitalization, as those who have been taken care of by carrying out mental health visits after medical hospitalization is associated with decreased readmissions.

The present study presents some limitations. The information on comorbidities, based on ICD-9-CM codes, does not consider the severity of the disease. Advanced stages of diseases are more likely to lead to unplanned hospitalizations compared to comorbidities at their earlier phases [29]. While the collection of such information at the patient level would enhance the model, it would also be extremely time-consuming. On the other hand, ICD-9-CM is a widely used and efficient system in many epidemiological studies [30]. Furthermore, administrative data may also be affected by some under-reporting or misdiagnosis, particularly for mental health conditions. Nevertheless, ministerial guidelines and recurrent training aimed to standardize coding, and ex-post checks are performed to further improve data quality. Similarly to the severity of the disease, additional information on drug therapies and outpatient visits could have helped in further characterizing the unplanned admission phenomenon. However, these data are usually managed by primary care physicians and the collection of this information would have been resource consuming. Finally, the study uses a binary variable for mental health conditions, but this may oversimplify the complexity of comorbid mental illnesses.

Despite these limitations, our study has several strengths. To the best of our knowledge, this is the first study conducted in Europe to evaluate the impact of mental health on unplanned hospitalizations using hospital discharge records (HDRs). The use of a standardized source such as HDR from a large sample (the entire population of an Italian Province) during a long study period makes results generalizable to the entire Italian population. HDRs represent a solid data source frequently used in the evaluation of healthcare services that can provide reliable estimates. In addition, the use of a standardized deprivation index made the analysis more robust, considering also socio-economic factors leading both to hospitalization and mental illness. Since our data covers a 7-year period, the effect of event-based or seasonal fluctuation on hospital admissions is limited, thus resulting in more stable estimates.

## 5. Conclusions

In a healthcare context increasingly constrained by limited financial and human resources, our findings highlight the significant impact of mental health comorbidities on unplanned hospitalizations. The strong association between mental disorders and unplanned hospitalizations, independent of socio-economic status and physical comorbidities, underscores the need for targeted strategies to improve the integrated management of mental and physical health conditions, mitigate risk for patients, and reduce expenditures at the National Health System.

Public health interventions, such as assertive community treatment or home-based care services with multidisciplinary teams, could help reduce the burden of unplanned hospital admissions by ensuring more effective and sustainable healthcare delivery. Furthermore, strengthening coordination between mental health services and primary care could facilitate early diagnosis and better management of psychiatric conditions, thereby improving clinical outcomes and alleviating the strain on healthcare systems.

Additional studies are needed to validate these findings in other regions and to identify the most cost-effective interventions. Integrating mental health into chronic disease prevention and management strategies should be a priority to enhance patient outcomes and reduce healthcare disparities.

## Figures and Tables

**Table 1:** Table with the characteristics of the selected admissions.

		Overall	%	Unplanned Admission	%	Planned Admission	%
Sample		59,374		43,293		16,081	
Gender							
	M	25,805	43.5%	18,284	42.2%	7521	46.8%
	F	33,569	56.5%	25,009	57.8%	8560	53.2%
Age classes							
	18–44	18,001	30.3%	13,833	32.0%	4168	25.9%
	45–64	14,705	24.8%	9260	21.4%	5445	33.9%
	65–79	15,559	26.2%	10,507	24.3%	5052	31.4%
	80+	11,109	18.7%	9693	22.4%	1416	8.8%
Caranci’s deprivation index classes							
	1	1772	3.0%	1288	3.0%	484	3.0%
	2	7983	13.4%	5756	13.3%	2227	13.8%
	3	13,191	22.2%	9268	21.4%	3923	24.4%
	4	1743	2.9%	1311	3.0%	432	2.7%
	5	34,489	58.1%	25,524	59.0%	8965	55.7%
Physical comorbidities							
	0	35,357	59.5%	25,000	57.7%	10,357	64.4%
	1	12,553	21.1%	9230	21.3%	3323	20.7%
	2 or more	11,464	19.3%	9063	20.9%	2401	14.9%
Mental comorbidities							
	No	57,822	97.4%	41,811	96.6%	16,011	99.6%
	Yes	1552	2.6%	1482	3.4%	70	0.4%

**Table 2:** Logistic analysis results, UHA as dependent variable.

Variable	Odds	*p*	Upper IC95%	Lower IC95%
Female gender	1.09	<0.01	1.05	1.14
Age classes				
18–44	0.58	<0.01	0.54	0.62
45–64	0.26	<0.01	0.25	0.28
65–79	0.30	<0.01	0.28	0.32
80+	ref			
Caranci’s deprivation index classes				
1	0.93	0.207	0.83	1.04
2	0.89	<0.01	0.48	0.94
3	0.84	<0.01	0.81	0.88
4	1.05	0.32	0.94	1.18
5	ref			
Physical comorbidities				
0	ref			
1	1.23	<0.01	1.17	1.30
2 or more	1.65	<0.01	1.56	1.75
Mental comorbidities	9.85	<0.01	7.74	12.55

## Data Availability

Data are unavailable due to privacy restrictions.
